# Assessment of Plasma Sodium to Potassium Ratio, Renal Function, Markers of Oxidative Stress, Inflammation, and Endothelial Dysfunction in Nigerian Hypertensive Patients

**DOI:** 10.1155/2020/6365947

**Published:** 2020-12-07

**Authors:** Oloruntoba A. Ekun, Folasade Daniel, Philip Adebola, Adeola Ajibare, Oyeronke O. Ekun, Oluwafunso O Omogoroye, Oluwafemi S. Ilori, Bankole J. Oluwasayo, Mary F. Oshundun, Sade R. Oyegbami

**Affiliations:** ^1^Department of Medical Laboratory Science, College of Medicine, University of Lagos, Lagos, Nigeria; ^2^Department of Medicine, Lagos State University, College of Medicine, Ikeja, Lagos, Nigeria; ^3^Department of Medicine, Lagos State University, Teaching Hospital, Ikeja, Lagos, Nigeria; ^4^Lagos State Health Service Commission, Mushin General Hospital, Lagos, Nigeria

## Abstract

**Background:**

This study investigated plasma sodium/potassium ratio, markers of oxidative stress, renal function, and endothelial dysfunction in hypertensive Nigerians.

**Materials and Methods:**

Five hundred forty-nine volunteers consisting of three hundred and twenty-four hypertensive and two hundred twenty-five controls participated in this study. Blood samples were collected from the participants and were analyzed for electrolytes, markers of oxidative stress, endothelial dysfunction, renal function, and inflammation, using ion-selective electrodes, spectrophotometric, and enzyme-linked immunosorbent assay methods, respectively.

**Results:**

The mean systolic blood pressure, mean diastolic blood pressure, mean arterial blood pressure, and body mass index (BMI) were significantly elevated among the hypertensive group when compared with control (*p* < 0.001). The mean sodium increased, while potassium and bicarbonate (HCO_3_^−^) decreased (*p* < 0.001) in hypertensive volunteers. The sodium-potassium ratio (Na^+^/K^+^) and urea were raised (*p* < 0.001) in the hypertensive group when compared with the control. Glutathione, superoxide dismutase, nitric oxide (NO), and catalase were significantly reduced (*p* < 0.001) while malondialdehyde (MDA), high-sensitivity C-reactive protein (hs-CRP), and ferritin were raised significantly (*p* < 0.001) in hypertensive participants. The odds of hypertension and its complications increased (*p* < 0.001) with an increase in BMI, Na^+^/K^+^, hs-CRP, MDA, and ferritin and a decrease in estimated glomerular filtration rate (eGFR), glutathione, superoxide dismutase, and catalase.

**Conclusion:**

An increase in Na^+^/K^+^, urea, hs-CRP, ferritin, MDA, and BMI and a decrease in eGFR, glutathione, and superoxide dismutase were associated with an increased risk of hypertension complication. Abnormal values of markers of oxidative stress, inflammation, and endothelial function could impact deleterious effects on the cardiovascular system among hypertensive Nigerians. A decreased bicarbonate possibly suggests an occult acid-base imbalance among hypertensive volunteers.

## 1. Introduction

Hypertension is the most common noncommunicable disease and the most prevalent cardiovascular disease risk factor worldwide. The risk of cardiovascular disease, cerebrovascular disease, and death increases progressively with each millimeter of mercury increase in blood pressure [1]. A study has shown that more than forty percent (40–50%) of the African population (where this study took place) is affected by this disease and that it is a cause of morbidity and mortality among the African population [2]. A blood pressure value of 120/80 mmHg is considered normal, while in general practice, the level of blood pressure above which treatment of hypertension is indicated is set at 140/90 mm Hg [3].

Electrolytes and water are absolutely two of the most critical components of normal physiological function. They influence many aspects of body function, balance the amount of fluid inside and outside of cells throughout the body, and play a vital role in muscle contraction and heart function [4]. A previous study has suggested that sodium and potassium have a significant effect on blood pressure by multiple mechanisms [5]. Interaction between sodium and potassium plays a significant role in the development of essential high blood pressure [6]. Few previous studies have demonstrated an association between sodium intake and high blood pressure [7, 8]. Thus, the sodium-potassium ratio (Na^+^/K^+^) may be more associated with blood pressure outcomes than either Na^+^ or K^+^ [9, 10]. An association between sodium to potassium (Na^+^/K^+^) ratio and the incidence of high blood pressure has been reported [8, 11]. However, most of the published studies reported urinary Na^+^/K^+^ ratio; there is a paucity of data on plasma Na^+^/K^+^. Plasma Na^+^/K^+^ might provide additional information towards the effective management of high blood pressure. Hence, this study evaluated the plasma Na^+^/K^+^ ratio among Nigerian hypertensive volunteers.

Oxidative stress, on the other hand, is characterized by disproportionate interactions between cellular antioxidants and prooxidants (reactive oxygen species (ROS) and reactive nitrogen species (RNS)) [12]. It is a consequence of either excessive production of reactive oxygen species (ROS)/reactive nitrogen species (RNS) or loss of antioxidant defenses [12]. A study which used nonspecific markers of oxidative damage previously observed higher superoxide and hydrogen peroxide production in hypertensive individuals, which returned to levels observed for control volunteers after blood pressure reduction [13], thus suggesting that essential hypertension may be associated with greater than normal lipoperoxidation and an imbalance in antioxidant status. It is suggested that oxidative stress may be important in the pathogenesis of essential hypertension or arterial damage related to essential hypertension [14]. However, there is a need for more data on markers of oxidative damage among Nigerian hypertensive individuals to enhance a better understanding between hypertension and markers of oxidative damage among this group of volunteers.

Moreover, an association between hypertension and inflammation has been a subject of intense discussion and research previously [15]. However, it is unclear whether inflammation is predominately a cause or effect of hypertension. A previous study by Kotchen [16] suggested a possible link between inflammation and elevated blood pressure. The possible mechanisms for this link have been explained to include an imbalance between vasoconstrictors and vasodilators, amplified thrombogenesis and platelet activation, and perhaps a direct effect of inflammatory mediators [17, 18]. It has been suggested that C-reactive protein (CRP), an inflammatory cytokine, plays an important role in vascular inflammation and can directly decrease the production of nitric oxide, a vasodilator. Additionally, ferritin, another acute-phase reactant, has been reported elevated in some inflammatory conditions [19]. Whether this occurs in hypertension is subject to research. However, the relationship between body iron stores and blood pressure (BP) status has not been well established [20] even among the Nigerian population. A previous study reported that erythropoietin which is closely associated with iron or ferritin metabolism could increase oxidative stress and lead to the accentuation of high blood pressure [21]. This study, therefore, aims to assess plasma sodium to potassium ratio, markers of oxidative stress, renal function, and endothelial dysfunction among the hypertensive Nigerian population.

## 2. Materials and Methods

### 2.1. Ethical Consideration of Consent Documentation

Ethical approval was obtained from the Health Research and Ethics committee (HREC), Lagos State University Teaching Hospital (LASUTH), before embarking on this study. This study was in concord with the declaration of Helsinki as informed consent was sought and obtained before participants were recruited into this study.

### 2.2. Criteria for Selection

#### 2.2.1. Inclusion Criteria

Participants (male and female) who were 18 years and above, whose blood pressure has been measured repeatedly and was found to be ≥140/90 mmHg and were referred to the cardiology unit with an unknown cause of hypertension were included as the test group. Apparently, healthy volunteers from both sexes with normal blood pressure (≤120/80 mmHg) were randomly recruited from among the College of Medicine and Teaching Hospital staff. These volunteers served as a control group.

#### 2.2.2. Exclusion Criteria

Children below 18 years of age, pregnant women, and hypertensive individuals with other comorbidities such as cancer, diabetes mellitus and kidney failure (acute and chronic), and anaemia at the time of this study were all excluded. The haemoglobin concentration of all volunteers was determined and a cut-off of 120 g/L and 130 g/L (for female and male, respectively) [22] was the minimum haemoglobin used to rule out anaemia. Volunteers whose haemoglobin concentration fell below the cut-off were excluded from this study.

#### 2.2.3. Antihypertensive Medications of Test Participants

The participants selected for the study were on one or more of calcium channel blockers (CCB), angiotensin-converting enzyme inhibitors (ACE-I), or diuretic.

### 2.3. Blood Sampling

Venous blood samples were obtained from all participants into plain vacutainer, Lithium heparinized, and dipotassium ethylene di-amine tetra-acetic acid (K_2_EDTA) bottles. The samples in plain and heparinized bottles were centrifuged at 3000 rpm for 5 minutes; the sera and plasma samples were dispensed into separate Eppendorf tubes. The samples were then stored at −20°C until analysis. K_2_EDTA samples were well mixed and were used to determine the haematocrit and haemoglobin of all volunteers that participated in this study.

### 2.4. Methods

Malondialdehyde (MDA), an index of lipid peroxidation, was determined using the method of Buege and Aust [23]. Superoxide dismutase activity was determined by the method described by Sun and Zigma [24]. Catalase activity was determined according to the method of Sinha et al. [25]. The reduced glutathione (GSH) was estimated according to the method described by Sedlak and Lindsay [26]. Nitric oxide was determined using the Griess reaction assay [27]. Electrolytes were determined using an ion-selective electrode (SFR, 4000), urea and creatinine were determined using autoanalyser Cobas C-111, high sensitivity-C-reactive protein (hs-CRP) and ferritin were determined using enzyme-linked immunosorbent assay (ELISA), and haematocrit and haemoglobin were determined using Mindray 5300BC. The plasma sodium to potassium ratio was obtained through calculation. Estimated glomerular filtration rate (eGFR) was obtained using the modification of diet in renal disease (MDRD) equation [28].

### 2.5. Statistical Analysis

The mean age, body mass index, systolic, diastolic, and mean arterial blood pressure variables were compared between hypertensive and normotensive volunteers. The plasma sodium to potassium ratio, markers of renal function, oxidative stress parameters and markers of inflammation and endothelial dysfunction were compared between hypertensive and normotensive participants. Test of normality was conducted on all continuous variables using kurtosis, Shapiro Wilk and Kolmogorov–Smirnov test. Normally distributed continuous variables were presented as mean ± standard deviation and were analyzed using parametric independent *t*-test, while the non-normally (skewed) distributed continuous variables were presented as median and interquartile range and were analyzed using nonparametric Wilcoxon–Mann–Whitney *U* test method. The levels of associations were determined using Pearson's and Spearman's rank correlations. Multivariate analysis of logistic regression analysis was performed on continuous variables to predict the odds of hypertension and its associated complications using continuous independent variables. All data were analyzed using Statistics and Data Science (STATA version 16 (StataCorp) and SPSS (IBM, Chicago) version 23. The significant level of probability was set at *p* < 0.05.

## 3. Results

Table 1 presented the mean demographic features and the blood pressure measurement of the hypertensive participants. The mean age of the hypertensive group was 59 ± 12.08 years, while their mean body mass index was 28.42 ± 4.10 kg/m^2^. The systolic, diastolic, mean arterial blood pressure and pulse pressure in the hypertensive group were 135 ± 15 mmHg, 88 ± 9 mmHg, 104 ± 10 mmHg, and 47 ± 12 mmHg, respectively. However, the mean values of sodium, potassium, and plasma Na^+^/K^+^ in the hypertensive group (Table 2) were 136.86 ± 5.95 mmol/l, 3.38 ± 0.49 mmol/l, and 41.32 ± 6.17, respectively. The hypertensive group median value of urea was 3.83 mmol/l, creatinine was 106.08 *μ*mol/l, hs-CRP was 10.07 *μ*g/ml, and serum ferritin was 206 ng/ml.  Table 3 showed the median, interquartile ranges for markers of oxidative stress in hypertensive and control volunteers. The median values were MDA (10.69 and 3.69) (*μ*mol/ml), GSH (6.34 and 9.66) (*μ*mol/ml), SOD (62.93 and 101.34) (*μ*mol/ml/min), CAT (191.35 and 431.82) (*μ*mol/ml/min), and NO (10.78 and 34.68) (*μ*mol/dl), respectively. The Spearman rank correlation and Pearson's correlation coefficients of the parameters studied were presented in Table 4. Antioxidants correlated negatively with MDA. There was a negative association between bicarbonate and systolic blood pressure. The odds of hypertension using the independent continuous variables were determined using logistics regression analysis in Table 5. The results were presented as odds ratio (OR) and 95% confidence interval (CI). Body mass index was (OR: 1.11 and CI: 1.073–1.149), Na^+^/K^+^ was (OR: 1.078: CI: 1.044–1.112), urea was (OR: 1.038: CI: 1.0139–1.061), hs-CRP was (OR: 2.050; CI: 1.770–2.371); ferritin was (OR: 1.023 CI: 1.019–1.028), MDA: (OR: 1.56, CI: 1.449–1.679), GSH: (OR: 0.663; CI: 0.614–0.716), CAT: (OR: 0.994 CI: 0.993–0.995), and NO: (OR: 0.881, CI: 0.864–0.898).

## 4. Discussion

In this study, the levels of electrolytes, electrolytes ratio, markers of renal function, and oxidative stress, as well as endothelial dysfunction, were determined among hypertensive volunteers since studies have shown a strong link between oxidative stress and hypertension [12, 29]. The hypertensive and control groups studied (Table 1) belong to the same age group (*p* > 0.05). Among the volunteers, 34.57% and 39.11% were males in hypertensive and control groups, respectively, while the female participants for hypertensive and control were 65.34% and 60.89%, respectively. Based on this percentage distribution of the studied volunteers, it appears that the Nigerian women tend to seek medical attention more than men and are more receptive to participating in research studies than their Nigerian male counterparts. The mean weight and body mass index (BMI) of the hypertensive participants were statistically higher (*p* < 0.05) than the control group. This observation suggests a possible causal relationship between increased body mass index and hypertension [30]. There was a significant (*p* < 0.05) increase in the mean systolic, diastolic, and mean arterial blood and pulse pressure in the hypertensive group when compared with the control.

The mean sodium value in the hypertensive group was significantly (*p* < 0.05) higher than the control. This observation was similar to the previous studies by Iyalomhe et al. [31], Anand and Shrishti [32], who demonstrated a significant increase (*p* < 0.05) in Na^+^ and Cl^−^ values in hypertensive volunteers when compared with the control group. Furthermore, the hypertensive patients had a mean K^+^ value that was lower than control (*p* < 0.05) (Table 2). Iyalomhe et al. [31], Anand and Shristi [32], and Momtaz et al. [33] also showed a similar trend in their previous studies. There was a significant increase (*p* < 0.05) in the mean sodium to potassium ratio (Na^+^/K^+^) among the hypertensive group when compared with the control group. An increase in plasma Na^+^/K^+^ as observed in this study may be more strongly associated with significant risk (*p* < 0.05) of hypertension and its outcomes as suggested by the outcome of the multivariate analysis (Table 5) than either nutrient alone. The outcome of this study appears similar to a previously documented study [34]. A previous study by Perez and Chang [10] reported that increased Na^+^/K^+^ was strongly associated with blood pressure outcomes. On the other hand, bicarbonate (HCO_3_^−^) was significantly reduced (*p* < 0.05) in the hypertensive group (Table 2) when compared with control. Mandel et al. [35] in their study reported that higher plasma bicarbonate was associated with lower odds of developing hypertension and vice versa, thus suggesting an inverse association between hypertension and plasma bicarbonate levels. An assessment of markers of renal function and estimated glomerular filtration rate (eGFR) among hypertensive volunteers showed that urea was higher (*p* < 0.05) among the hypertensive group while creatinine and eGFR values were similar (*p* > 0.05) among hypertensive and normotensive control groups studied. However, a higher eGFR was observed to be associated with lower odds of developing hypertension as observed from the multivariate analysis studied (Table 5) [36]. However, our observation with respect to renal function among the hypertensive volunteers was at variance with previous studies [37, 38] that reported occult renal dysfunction in their study. The possible reason for this could be associated with effective control of the blood pressure among the majority of the hypertensive volunteers who participated in this study.

Furthermore, we observed a significant increase (*p* < 0.05) in the serum hs-CRP and ferritin in the hypertensive group studied (Table 2). This observation suggests a causal relationship between inflammation and high blood pressure. Our finding with respect to hs-CRP was similar to previous studies by Lloyd-Jones et al. [17], Adams [19], Galan et al. [20], Tofano et al. [39], and Wium-Andersen et al. [40]. Low-grade inflammatory process may be a factor for the pathogenesis of essential hypertension and predicting the risk of future cardiovascular events and target organ damage in hypertensive individuals [40, 41]. This observation is also in concord with the previous study by Tofano et al. [39] that suggested that variation of circulatory high sensitive C-reactive protein (hs-CRP) reflects the chronic inflammatory process and is associated with essential hypertension and predicts cardiovascular outcome. Moreover, another study suggested that elevation of hs-CRP in the normotensive individuals could be associated with the risk of developing hypertension later in life [42]. A significant elevation in the ferritin level among hypertensive volunteers studied could expose them to severe hypertensive outcomes; this is because ferritin metabolism can increase oxidative stress, thus leading to the accentuation of hypertension [21]. Increased serum ferritin levels and the prevalence of hyperferritinemia in the hypertensive group may reflect another underlying mechanism for the development and or worsening of hypertension [43].

In addition to this, an evaluation of oxidative stress markers among the participants in this study showed that MDA was significantly (*p* < 0.05) elevated among hypertensive volunteers (Table 3) when compared with the normotensive control, whereas glutathione (GSH) and antioxidant enzymes (CAT, SOD) were significantly lower (*p* < 0.05) among hypertensive individuals. An increase in the level of MDA and a decrease in antioxidant defense enzymes as observed in this study suggest a higher lipid peroxidation and oxidative stress state. Observation from this study is in harmony with previous studies by Ahmad et al. [12] Kachhawa et al. [29], and Rodrigo et al. [44], who reported that essential hypertension was associated with greater than normal level of MDA and by extension greater than normal level of lipoperoxidation and an imbalance in antioxidant, and antioxidant enzyme status, suggesting that oxidative stress may be important in the pathogenesis and worsening of essential hypertension or in arterial damage related to essential hypertension [45,46]. Thus, excessive lipid peroxidation which probably occurred among the hypertensive group might have led to a disproportionate generation of reactive oxygen species (ROS) concentration leading to an imbalance in free radicals as a result of inadequate cellular antioxidant defense to completely inactivate the reactive oxygen species (ROS) and reactive nitrogen species (RNS) generated [12]. Animal studies have shown that an increase in blood pressure is associated with increased oxidative stress [12, 47]. In this study, nitric oxide (NO) also showed a significantly (*p* < 0.05) lower value among hypertensive individuals when compared with the normotensive control group. A diminished nitric oxide as observed in this study suggests an endothelial dysfunction among the hypertensive group as nitric oxide is majorly derived from vascular endothelium. Our study with regard to NO is in agreement with the previous study by Tsikas et al. [48] who also documented lower nitric oxide value thus suggesting that lower nitric oxide may play an important pathophysiological role in the development of hypertension and its progression. This may be due, in large part, to O^2−^ excess and decreased NO bioavailability in the vasculature and kidneys, and to ROS-mediated cardiovascular remodeling. Thus, oxidative excess in hypertensive patients leads to diminished NO [46, 49] and correlates with the degree of impairment of endothelium-dependent vasodilation and with cardiovascular events [50].

The level of association of the biochemical and oxidative stress markers among the hypertensive group (Table 4) showed that all the endogenous antioxidant and antioxidant enzymes studied correlated positively (*p* < 0.05) with each other but correlated negatively (*p* < 0.05) with the Malondialdehyde (MDA) among the hypertensive group studied. This observation corroborates the previous studies by Redon et al. [51] and Tanito et al. [52] who reported that hypertension was associated with excessive amounts of ROS in humans and various animal models of hypertension. An increased amount of ROS among human and experimental animal hypertension has been associated with diminished antioxidant status [53]. Thus, an increase in Malondialdehyde was associated with a decrease in all the endogenous antioxidant markers studied. Endogenous antioxidants correlated negatively with the systolic blood pressure; however, this association was insignificant. There was a significant (*p* < 0.05) negative association between nitric oxide and systolic blood pressure, thus suggesting that hypertension possibly precipitates diminished nitric oxide and vice versa. A similar association between hypertension and nitric oxide has been observed previously [54]. Systolic blood pressure also correlated negatively (*p* < 0.05) with plasma bicarbonate. This observation suggests that hypertension may impact a deleterious effect on acid-base homeostasis and possibly increase the anion gap with a possible consequence of metabolic acidosis. Our observation in this regard agrees with the previous pieces of literature [55, 56].

A multivariate logistic regression (Table 5) analysis indicated that a significant (*p* < 0.05) odds of hypertension increases with an increase in the plasma sodium-potassium ratio (Na^+^/K^+^) [36], MDA, hs-CRP, ferritin, weight, and body mass index. The odds of hypertension were found to be increased with a decrease in catalase, nitric oxide, glutathione, and superoxide dismutase, thus suggesting important roles that could be played by electrolyte imbalance [4], inflammation [42], and oxidative stress in the pathogenesis and highlighting of high blood pressure.

## 5. Conclusion

To the best of our knowledge, this appears to be the first time that the plasma sodium to potassium ratio will be assessed among hypertensive individuals. There is a need for more studies on this for a better understanding of any possible diagnostic and clinical utility of this ratio. Our study also showed that low plasma bicarbonate was common among hypertensive Nigerians. Hypertension among the Nigerian population appears to be associated with some degree of oxidative stress and diminished nitric oxide synthesis even when the blood pressure appears to be controlled. There may be a need to critically mitigate oxidative stress and diminished nitric oxide synthesis to optimize the full gains of their management.

## Figures and Tables

**Table 1:** Sociodemographic characteristics of volunteers.

Variables	Hypertension(*n* = 324) mean ± SD	Control(*n* = 225) mean ± SD	*p* value
Sex	Male: 112 (34.57%)	Male: 88 (39.11%)	
	Female: 212 (65.34%)	Female: 137 (60.89%)	
Age (yrs)	54.53 ± 12.08	53.57 ± 4.4	0.135
Height (m)	1.66 ± 0.09	1.69 ± 0.09	0.003^*∗*^
Weight (kg)	75.05 ± 15.00	67.51 ± 13.77	<0.001^*∗*^
BMI (kg/m^2^)	27.20 ± 5.67	23.94 ± 5.90	<0.001^*∗*^
Systolic Bp (mmHg)	135 ± 15	120 ± 10	<0.001^*∗*^
Diastolic Bp (mmHg)	88 ± 9	79 ± 9	<0.001^*∗*^
Mean arterial pressure (mmHg)	104 ± 10	92 ± 9	<0.001^*∗*^
Pulse pressure (mmHg)	47 ± 12	41 ± 9	<0.001^*∗*^

^*∗*^Significant probability (*p* < 0.05). Bp: blood pressure; mmHg: millimeters of mercury.

**Table 2:** Biomarkers of renal function, plasma sodium-potassium ratio, and inflammatory biomarkers between hypertensive and control volunteers.

Parameter	Hypertension (*n* = 323) mean ± SD	Control (*n* = 225) mean ± SD	*p* value
Uric acid (*μ*mol/l)	396.3 ± 121.6	402.9 ± 129.2	0.7600
Na^+^(mmol/L)	136.86 ± 5.95	132.22 ± 4.54	<0.001^*∗*^
K^+^(mmol/L)	3.38 ± 0.49	3.50 ± 0.58	0.0100^*∗*^
Plasma Na^+^/K^+^	41.32 ± 6.17	38.66 ± 5.90	<0.001^*∗*^
HCO_3_^−^ (mmol/L)	19.58 ± 3.57	22.93 ± 3.23	<0.001^*∗*^
Urea (mmol/L) (interquartile range)	3.83 (3.16–4.66)	3.66 (3.16–4.16)	0.0495^*∗*^
Creatinine (*μ*mol/L) (interquartile range)	79.56 (63.65–97.24)	79.56 (70.72–88.40)	0.9693
eGFR (ml/min/1.73 m^2^) (interquartile range)	88.019 (74.85–108.26)	92.399 (77.41–105.42)	0.2560
hs-CRP (*μ*g/mL) (interquartile range)	10.07 (4.12–12.52)	1.24 (0.76–2.20)	<0.001^*∗*^
CAT (*μ*mol/ml/min) (interquartile range)	206 (148–297.14)	107.20 (75.44–134)	<0.001^*∗*^

^*∗*^Significant probability (*p* < 0.05).

**Table 3:** Biomarkers of oxidative stress and endothelial function of hypertensive and control volunteers.

Parameter	Hypertension (*n* = 320) median (IQR)	Control (*n* = 225) median (IQR)	*p* value
GSH (*μ*mol/ml) (interquartile range)	6.34 (3.99–9.02)	9.66 (8.23–11.55)	<0.001^*∗*^
MDA (*μ*mol/ml) (interquartile range)	10.69 (6.27–11.85)	3.69 (2.23–11.55)	<0.001^*∗*^
NO (*μ*mol/dl) (interquartile range)	10.78 (7.58–16.12)	34.68 (31.07–37.53)	<0.001^*∗*^
SOD (*μ*mol/ml/min) (interquartile range)	62.93 (30.40–87.87)	101.34 (92.08–107.69)	<0.001^*∗*^
CAT (*μ*mol/ml/min) (interquartile range)	191.35 (93.11–310.09)	431.82 (274.32–517.67)	<0.001^*∗*^

^*∗*^Significant probability (*p* < 0.05); IQR: interquartile range.

**Table 4:** Spearman rank correlation and Pearson's correlation coefficient studies of the biochemical parameters studied in hypertensive volunteers.

Variables (*n* = 323)	*r*_s_	*p* value
Superoxide dismutase-catalase	0.2114	<0.001^*∗*^
Urea-creatinine	0.3699	<0.001^*∗*^
Glutathione-ferritin	−0.1627	0.0034^*∗*^
Malondialdehyde-glutathione	−0.3965	<0.001^*∗*^
Malondialdehyde-ferritin	0.1884	<0.001^*∗*^
Nitric oxide-glutathione	0.3904	<0.001^*∗*^
Nitric oxide-malondialdehyde	−0.3532	<0.001^*∗*^
Superoxide dismutase-nitric oxide	0.3257	<0.001^*∗*^
Superoxide dismutase-malondialdehyde	−0.3388	<0.001^*∗*^
Superoxide dismutase-glutathione	0.3223	<0.001^*∗*^
Superoxide dismutase-ferritin	−0.1303	0.0190^*∗*^
Catalase-glutathione	0.3156	<0.001^*∗*^
Catalase-malondialdehyde	−0.3949	<0.001^*∗*^
Catalase-nitric oxide	0.3637	<0.001^*∗*^
Urea-hs-CRP	0.1290	0.0204^*∗*^
hs-CRP-diastolic blood pressure	0.1654	0.0029^*∗*^
Superoxide dismutase-systolic blood pressure	−0.0948	0.0891
Catalase-systolic blood pressure	−0.0141	0.8006
Nitric oxide-systolic blood pressure	−0.1158	0.0376^*∗*^
Glutathione-systolic blood pressure	−0.0760	0.1729
Malondialdehyde-systolic blood pressure	0.0171	0.7600

	*r*	*p* value
BMI-weight	0.8643	<0.001^*∗*^
BMI-systolic blood pressure	0.1440	0.0094^*∗*^
Systolic blood pressure-HCO_3_^−^	−0.1132	0.0429^*∗*^
Systolic-diastolic blood pressure	0.5776	<0.001^*∗*^

^*∗*^Significant probability (*p* < 0.05).

**Table 5:** Logistic regression analysis on independent variables predicting the odds of hypertension and its complications.

Variables	Odds ratio	Std error	*p* value	95% confidence interval
Weight (kg)	1.038	0.006	<0.001^*∗*^	1.025–1.052
Body mass index (kg/m^2^)	1.111	0.019	<0.001^*∗*^	1.073–1.149
Na^+^/K^+^	1.078	0.017	<0.001^*∗*^	1.044–1.112
Urea (mmol/L)	1.038	0.012	0.0020^*∗*^	1.0139–1.061
eGFR (mL/min/1.73 m^2^)	0.9931	0.0025	0.0080^*∗*^	0.988–0.998
hs-CRP (*μ*g/ml)	2.050	0.152	<0.001^*∗*^	1.772–2.371
Ferritin (ng/ml)	1.023	0.002	<0.001^*∗*^	1.019–1.028
GSH (*μ*mol/ml)	0.663	0.126	<0.001^*∗*^	0.614–0.716
SOD (*μ*mol/ml/min)	0.963	0.004	<0.001^*∗*^	0.956–0.971
CAT (*μ*mol/ml/min)	0.994	0.006	<0.001^*∗*^	0.993–0.995
NO (*μ*mol/dl)	0.881	0.008	<0.001^*∗*^	0.864–0.898
MDA (*μ*mol/ml)	1.560	0.058	<0.001^*∗*^	1.449–1.679

^*∗*^Significant probability (*p* < 0.05).

## Data Availability

The datasets regarding this study can be obtained from the corresponding author upon reasonable request.
